# A Hyperspectral Data 3D Convolutional Neural Network Classification Model for Diagnosis of Gray Mold Disease in Strawberry Leaves

**DOI:** 10.3389/fpls.2022.837020

**Published:** 2022-03-11

**Authors:** Dae-Hyun Jung, Jeong Do Kim, Ho-Youn Kim, Taek Sung Lee, Hyoung Seok Kim, Soo Hyun Park

**Affiliations:** Smart Farm Research Center, Institute of Science and Technology (KIST), Gangneung-si, South Korea

**Keywords:** hyperspectral imaging, strawberry gray mold, early diagnosis, asymptomatic infection, 3D convolutional neural network

## Abstract

Gray mold disease is one of the most frequently occurring diseases in strawberries. Given that it spreads rapidly, rapid countermeasures are necessary through the development of early diagnosis technology. In this study, hyperspectral images of strawberry leaves that were inoculated with gray mold fungus to cause disease were taken; these images were classified into healthy and infected areas as seen by the naked eye. The areas where the infection spread after time elapsed were classified as the asymptomatic class. Square regions of interest (ROIs) with a dimensionality of 16 × 16 × 150 were acquired as training data, including infected, asymptomatic, and healthy areas. Then, 2D and 3D data were used in the development of a convolutional neural network (CNN) classification model. An effective wavelength analysis was performed before the development of the CNN model. Further, the classification model that was developed with 2D training data showed a classification accuracy of 0.74, while the model that used 3D data acquired an accuracy of 0.84; this indicated that the 3D data produced slightly better performance. When performing classification between healthy and asymptomatic areas for developing early diagnosis technology, the two CNN models showed a classification accuracy of 0.73 with regards to the asymptomatic ones. To increase accuracy in classifying asymptomatic areas, a model was developed by smoothing the spectrum data and expanding the first and second derivatives; the results showed that it was possible to increase the asymptomatic classification accuracy to 0.77 and reduce the misclassification of asymptomatic areas as healthy areas. Based on these results, it is concluded that the proposed 3D CNN classification model can be used as an early diagnosis sensor of gray mold diseases since it produces immediate on-site analysis results of hyperspectral images of leaves.

## Introduction

The strawberry (*Fragaria ananassa*) is a widely grown species in the Fragaria genus, and it is produced and consumed throughout the world. In 2021, the global fresh strawberry market was estimated to be 1.772 billion dollars, making it an important crop production system. In South Korea, the total production value of strawberries is 1.34 trillion South Korean won, which is the largest market among horticultural crops. However, the gray mold disease, which is caused by *Botrytis cinerea*, occurs in an average of 10–15% of strawberry-cultivated land each year, resulting in an economic damage of 110 billion South Korean won. When the disease occurs, many spores are formed in the lesion and propagate to other places, spreading the disease and increasing the damage. Therefore, it is necessary to diagnose and contain the disease at its early stages (Back et al., 2014; Hao et al., 2017).

The gray mold disease fungus (*Botrytis cinerea*) is a pathogenic fungus that damages a wide variety of hosts including strawberries (*Fragaria* × *ananassa*), grapes (Vitis vinifera), cucumbers (*Cucumis sativus*), and tomatoes (*Solanum lycopersicum*). As such, it is known as a disease that causes significant losses of many economic crops (Forges et al., 2018). The disease occurs more often in excessively humid environments (Fanourakis et al., 2013). The symptoms occur on the crop’s flowers, leaves, stems, and even the fruits, affecting seriously the growth of the crops and reducing yields. In gray mold disease, various symptoms are observed before clear signs of a diseased plant appear (Awad, 2017). In strawberries, brown symptoms appear on leaves and petals, while the symptoms in fruits are browning and softness. In addition, the gray mold disease latent infections are not visible in cultivation but become evident later on during the postharvest chain. The most efficient way to block the propagation of the gray mold pathogenic fungus is to apply fungicides to the infected areas; however, if these are used excessively, it may incur not only a high cost, but also environmental and health problems for workers (Xie et al., 2017). Therefore, if the occurrence of the pathogenic fungus can be diagnosed at an early stage, it may be possible to manage the disease with minimal fungicide applications.

To detect gray mold disease, traditional immunological detection methods such as enzyme-linked immunosorbent assays (ELISA), polymerase chain reaction (PCR), and tissue blotting are accurate (Meyer et al., 2000; Govrin et al., 2006; Fahrentrapp et al., 2019). Yet, these methods collect samples in a destructive manner, and they require skilled labor, costly equipment, and a great deal of time. As such, there is a need for rapid sensing and analysis technology to identify and respond to crop infections that are grown in protected environments such as greenhouses.

Currently, non-destructive measurement technologies are developing rapidly and are being used for a variety of purposes in the field of agriculture. Typical areas where agricultural non-destructive sensing is used can be divided into the field of large-scale remote sensing, which measures large areas for crop yield status, soil nutrient status and droughts, and the field of detailed analysis, which determines the state of plants and diagnoses diseases in detail. In detailed analysis sensing technology, there have been many studies recently that used hyperspectral imaging (HSI) (Cen et al., 2016; Lowe et al., 2017; Golhani et al., 2018; Zhang et al., 2020). This is because HSI is capable of collecting spectral information on hundreds of wavelength bands for each pixel based on image data, and high resolution devices are becoming common. This technology is actively being used for diagnosing the status and symptoms of diseases in plants (Bock et al., 2010; Gao et al., 2020). Zhu et al. (2017) used hyperspectral imaging data to examine short-term (48 h) tobacco mosaic virus infections. They performed rapid preprocessing through selecting effective wavelengths (EWs) and proposed a neural network structure classification model, reporting an accuracy of 0.95. Gao et al. (2020) examined the possibility of using HSI for non-destructive detection of asymptomatic and symptomatic stages of a virus (GLRaV-3) in the grapevine leaves of red wine grapes. In this way, the feasibility of using hyperspectral images for plant disease prediction has already been proven; additionally, there are many studies underway to increase its applicability in the field.

Machine learning and deep learning techniques have advantages in processing large amounts of data and are widely applied in analyzing wavelength data for each pixel of hyperspectral images. In the first half of the 2010s, there were studies that processed training data or applied classification models to images through employing certain feature information such as partial least squares regression (PLSR), principal component regression (PCR) (Zhao et al., 2017), support vector machines (SVMs), and artificial neural networks (ANNs) (Schmidhuber, 2015; Bishop et al., 2019; Jung et al., 2021). Since 2015, more in-depth learning has become possible by endowing graphics processing units (GPUs) with the computing power to rapidly process large amounts of information; this approach is still used in many fields under the name deep learning (Kamilaris and Prenafeta-Boldú, 2018; Feng et al., 2020). Because of hyperspectral data containing hundreds of reflectance data for each of the high-resolution pixels, a single image includes tens of millions of data, and the use of deep learning technology is essential for handling them without processing. In the last decade, CNNs have been increasingly employed in plant phenotyping community. They have been very effective in modeling complicated concepts, owing to their ability of distinguishing patterns and extracting regularities from data. Examples include variety identification in seeds (Taheri-Garavand et al., 2021b) and in intact plants by using leaves (Nasiri et al., 2021). Among the 2D CNN models, model structures such as GoogLeNet, Resnet, and Fast-RCNN, which have been used extensively in image classification and have been verified in terms of performance, are often used in the development of classification models for hyperspectral data (Saleem et al., 2019).

Given that hyperspectral images are 3D hypercubes with inherent spectral and spatial continuity, a 3D CNN method that integrates spectral and spatial information is a more suitable model. In the field of magnetic resonance imaging (MRI), which are image data with a similar 3D cube structure, studies on the use of 3D CNNs have already been published (Zou et al., 2017; Tang et al., 2021). The 3D CNN approach has the advantage of simultaneously processing 3D regions that have shared spatial-spectral data; therefore, it has been reported to be useful for hyperspectral data as well (Yu et al., 2020). Al-Sarayreh et al. (2020) also reported on the feasibility of obtaining an improved accuracy rate of 96.9% by using a 3D CNN to classify red meat.

The present study proposes a deep learning model for classifying infected, asymptomatic infected, and non-infected areas among local areas using hyperspectral images in order to perform early diagnosis of gray mold disease in strawberries. Specifically, it introduces a method that can extract regions of interest (ROIs) for areas that show symptoms seen by the naked eye after inducing gray mold disease in the leaves of actual strawberries that are being cultivated, as well as asymptomatic areas that show no obvious symptoms but have the disease 24 h later. A 3D CNN classification model that uses collected hyperspectral image data is proposed in this study, and its performance improvements are verified through a comparison with 2D CNN models.

## Materials and Methods

### Individual Strawberry Samples and Gray Mold Inoculation

**FIGURE 1:**
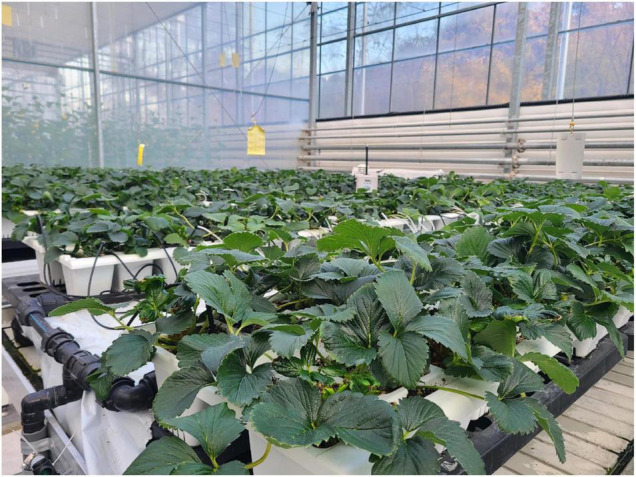
Strawberry cultivation before gray mold disease inoculation.

This study selected the “Seolhyang” strawberry as its target crop, which is a variety mainly cultivated in Korea. The plants were grown *via* hydroponics in a greenhouse (Figure 1). The strawberry samples in the early stages, i.e., around 1–3 weeks after planting, were used for the experiments. After being inoculated with gray mold, they were stored separately in chambers to induce the onset of the disease.

**FIGURE 2:**
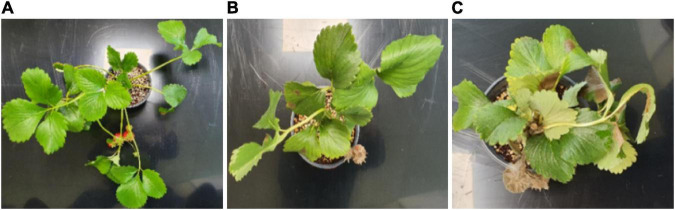
Onset of disease after gray mold disease inoculation [**(A)** healthy state before inoculation, **(B)** moderate stage 2 days after inoculation, **(C)** severe stage 4 days after inoculation].

A small amount of gray mold disease fungus mycelium was inoculated in the center of a potato dextrose agar (PDA) medium and incubated in the dark for 5 days at 20°C. Then, it was moved to a light/dark incubator and irradiated with light for 16 h per day to induce spore formation. The spores that formed on the PDA medium were harvested with a sterilized KH2PO4-glucose (0.05% Tween-20) solution, and sterilized gauze was used to remove the mycelium. The spores were counted with a hemocytometer, and the KH2PO4-glucose (0.05% Tween-20) solution was used to adjust the mixture to 1 × 106 conidia/mL. The prepared suspension was used to spray-inoculate the strawberry plants, and they were incubated at 20°C for 3–4 days in a humid room to induce the onset of the disease. As shown in Figure 2, 50 healthy samples without disease were prepared separately, which were sprayed with a solution that did not contain the mycelium. From 100 disease-induced samples, leaves with moderate and severe infection were collected. The strawberry leaves for the measurements included 100 leaves from healthy areas (where the disease did not occur) and 150 leaves where the disease occurred.

### Collection of Hyperspectral Imaging Data

**FIGURE 3:**
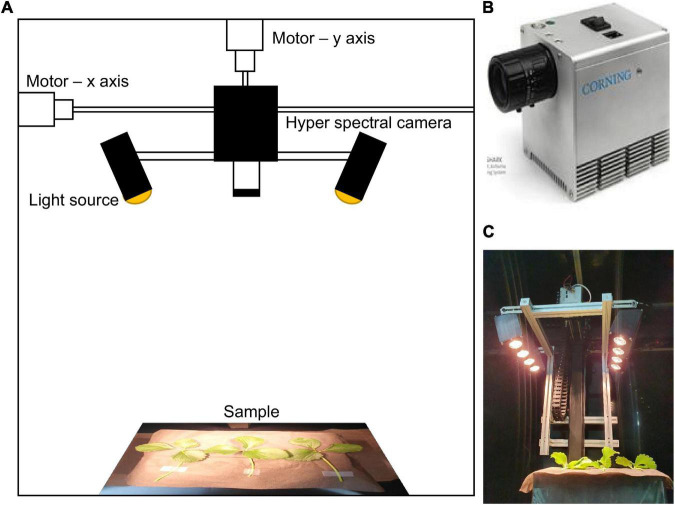
**(A)** Hyperspectral measurement system overview, **(B)** hyperspectral camera equipment, and **(C)** hyperspectral image measurement.

The hyperspectral imaging data measurements were performed in a dark chamber equipped with a microHSI™ (Corning Inc., United States) and a moveable stage as shown in Figure 3. A hyperspectral camera was installed in the middle of the upper part, and a conveyor belt and motor were installed along the x- and y-axes to allow the camera to move. The y-axis is the distance between samples; in the experiments, the distance between the lens and samples was fixed at 500 mm, taking into account the focal point and resolution. The total length of the x-axis was 1,500 mm, and in the experiments, the camera moved 300 mm at a rate of 150 mm/s and captured line scan images. The hyperspectral images were set to have a precision of 150 wavelengths in the visible-near-infrared wavelength band of 400–1,000 nm. As the light source, four 20 W halogen lamps were installed along the x-axis on both sides (for a total of 8) in order to illuminate the scan line (Figure 3C). The size of the captured hyperspectral images was 682 × 1,497, and 3–5 strawberry leaves were measured per each round of scanning. The Spectral library in the Python 3.8 environment was used to analyze the measured hyperspectral imaging data.

### Selection of Training Data Regions of Interest

**FIGURE 4:**
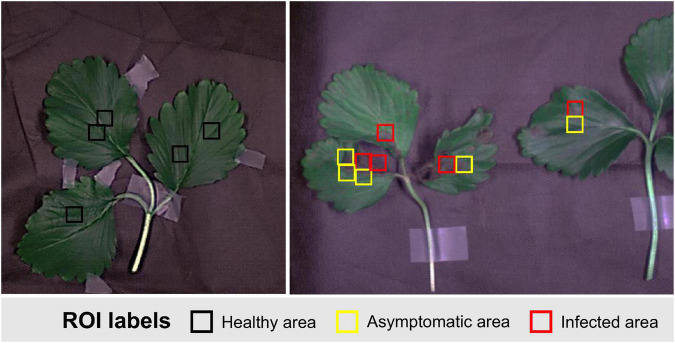
Example of ROI selection for classification of strawberry gray mold disease.

In this study, a hyperspectral imaging data set was first acquired in order to classify gray mold disease occurring on strawberry leaves. The areas where symptoms definitely appeared in the inoculated strawberry leaves were defined as infected areas, and the areas where the fungal disease had spread from the infected site within 2 days but showed no visible symptoms were defined as asymptomatic areas. Areas that looked healthy in the leaves where the disease did not occur were defined as healthy areas (Figure 4). For the ROIs, a dedicated extraction program was used to convert the hyperspectral images to RGB images, and then, select square areas of 16 × 16 pixels manually. 1,056 ROIs were collected for infected areas, 696 for asymptomatic areas, and 1,358 for healthy areas.

### 2D and 3D Convolutional Neural Network Classification Model Development

#### 2D Convolutional Neural Network Model

**FIGURE 5:**
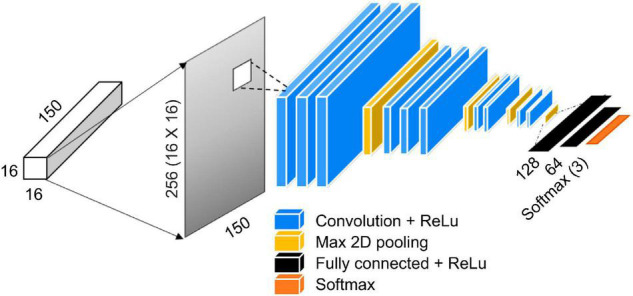
Overview of CNN model usage after converting 3D data to 2D data.

2D CNN models exhibit groundbreaking performance that surpasses conventional image processing techniques in object recognition and classification of RGB images (Rawat and Wang, 2017). It has been shown that the algorithms used for RGB images are effective in processing large amounts of data even in hyperspectral images. This study also used three types of 2D CNN models. In order to use the local area data of the previously extracted leaves in a 2D CNN, the 16 × 16 × 150 3D hypercube data were converted into 256 × 150 2D planar data based on the pixels in the vertical direction As shown in Figure 5, this study developed a model by configuring the 2D CNN model as layers. The shape and specifications of the model are shown in Tables 1, 2. The 2D and 3D CNN models in this study were developed based on the Tensorflow and Keras libraries in Python 3.8. As a GPU, this study used an NVIDIA RTX3090 equipped with 24 GB memory.

**TABLE 1:** 2D CNN model architecture.

Layer (Type)	Filter (kernel)	Output Shape	Parameters
Conv 2D-1	128 (4, 4)	(253, 147, 128)	2,176
Conv 2D-2	128 (4, 4)	(250, 144, 128)	262,272
Max pooling 2D	(2, 2)	(125, 72, 128)	0
Dropout	30%	(125, 72, 128)	0
Conv 2D-3	64 (3, 3)	(122, 69, 64)	13,136
Conv 2D-4	64 (3, 3)	(119, 66, 64)	6,560
Max pooling 2D	(4, 4)	(29, 16, 64)	0
Dropout	30%	(29, 16, 64)	0
Conv 2D-5	64 (3, 3)	(26, 13, 64)	65,600
Max pooling 2D	(2, 2)	(13, 6, 64)	0
Dropout	30%	(13, 6, 64)	0
Conv 2D-6	32 (3, 3)	(10, 3, 32)	32,800
Flatten	960		0
Dense-1	128		32,896
Dense-2	64		14,958
Dense-3	3		156

**TABLE 2:** 3D CNN model architecture.

Layer (Type)	Filter (kernel)	Output Shape	Parameters
Conv 3D-1	64 (2, 2, 8)	(15, 15, 135, 64)	4,160
Max pooling 3D	(2, 2, 2)	(7, 7, 67, 64)	0
Batch normalization		(7, 7, 67, 64)	256
Conv 3D-2	64 (2, 2, 8)	(6, 6, 60, 64)	131,136
Batch normalization		(6, 6, 60, 64)	256
Conv 3D-3	128 (2, 2, 4)	(5, 5, 57, 128)	131,200
Batch normalization		(5, 5, 57, 128)	512
Conv 3D-4	256 (2, 2, 2)	(4, 4, 56, 256)	262,400
Max pooling 3D	(1, 1, 2)	(2, 2, 56, 256)	0
Batch normalization		(2, 2, 28, 256)	1,024
Global average pooling 3D	256		0
Dense-1	512		131,584
Dense-2	1		513

#### Use of 2D Convolutional Neural Network-Based Resnet and VGGnet

**FIGURE 6:**
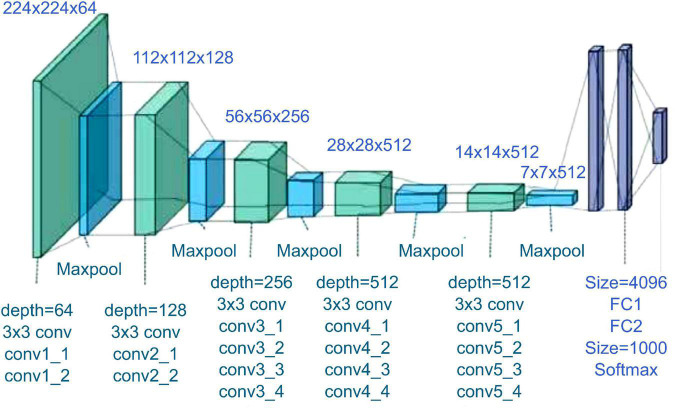
VGGnet-19 architecture (Zheng et al., 2018).

2D CNN models show high reliability among the reported models in many studies (He et al., 2016; Yu et al., 2019); two models that are used in various industries were selected and used as comparison groups in this study. The same input and output as in the previously proposed 2D CNN were used. The first 2D CNN algorithm that was selected as a comparison target is VGGnet-19 (Figure 6). VGGNet is a model proposed by Simonyan and Zisserman (2014), and it came in second behind GoogLeNet. However, it is used more often because it is structurally simpler, easy to understand, and simple to test. In addition, previous studies have reported on the feasibility of using it on hyperspectral images to develop classification models (He et al., 2019).

**FIGURE 7:**
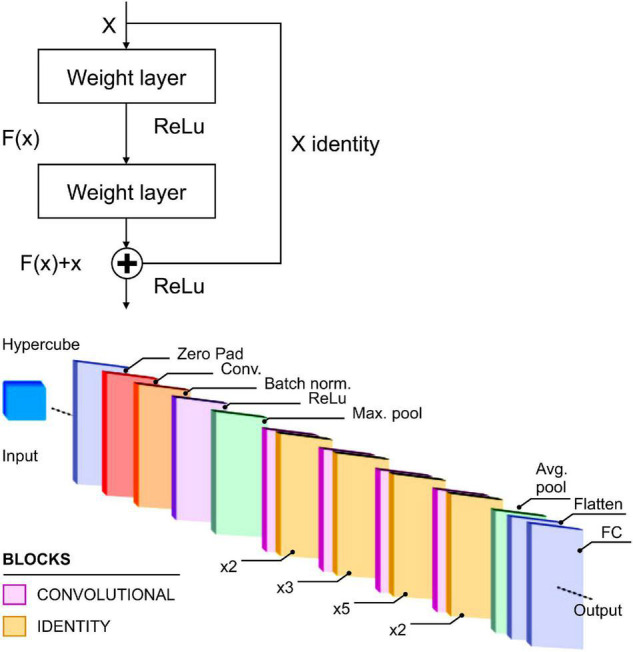
Residual block concept (left) and Resnet-50 architecture (right) (Medus et al., 2021).

The second comparison model is Resnet 50. The Resnet residual block is a structure that has different properties than a conventional CNN model, in which the input and output are connected as shown in Figure 7 (left). In these layers, there is a shortcut device, which adds the input values without modification to the output. Through this shortcut, the direct effects of the input values are transferred to the output without modification; thus, it is able to ameliorate the vanishing gradient problem that occurs when there are many layers among the deep convolutional layers (He et al., 2016).

#### 3D Convolutional Neural Network Model

A 3D CNN is an approach that uses 3D convolution calculations, and the filters of all convolution layers are created in three dimensions. In addition, the feature maps that are generated by each filter are also configured in 3D. Because of this structure, the spectral- temporal values of continuous pixels can easily be used for learning in 3D CNNs. Generally, in 3D convolutions, W × W × K hypercube data are received as input. Here, W × W is the image resolution, and K is the number of channels which corresponds to the spectral bandwidth. In addition, the filters that are used in 3D convolution calculations have an H × H × K size; the H × H is the filter’s horizontal and vertical dimensions, while the K is the pixels’ number of spectral channels. Like 2D convolutions, the convolution calculations are performed as the filter moves over the image like a scan; however, the movement occurs not only in the horizontal and vertical directions spatially, but also by the amount of the stride (L) along the wavelength band axis. Thus, in the *u^l^*_*i,j,k*_ that is calculated by 3D convolutions, the output that is generated from the input value *x*_*i+p, j+q, f+k*_ *via* the non-linear activation function can be calculated as $z_{i+p,j+q,f+k}^{\left(l-1\right)}$. The moveable distances along the 3D axes at this time are K, H, and H, and the movement occurs while scanning toward the locations of k, p, and g. *b*_*ijk*_ is the generated bias that can be expressed as follows:


(1) $$z_{i,j,k}^{l}=f\,\left(x\,l_{i+p,j+q,f+k}\right)$$

(2) $$u\,l_{i,j,k}=\sum_{k=0}^{K-1}\sum_{p=0}^{H-1}\sum_{q=0}^{H-1}z\,{\left(l-1\right)}_{i+p,j+q,f+k}+b_{i\,j\,k}$$

**FIGURE 8:**
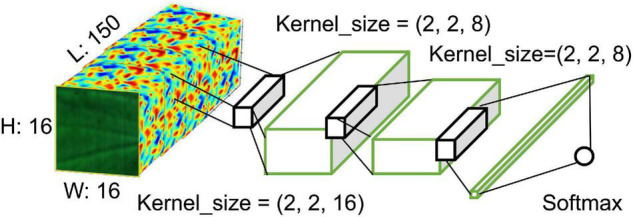
Proposed 3D CNN structure of this study.

The hypercube form of the input x is 16 × 16 × 150, and it was used in the model development *via* a 3D CNN with the structure shown in Figure 8.

#### 3D Convolutional Neural Network Model Based on Extended Input Data

**FIGURE 9:**
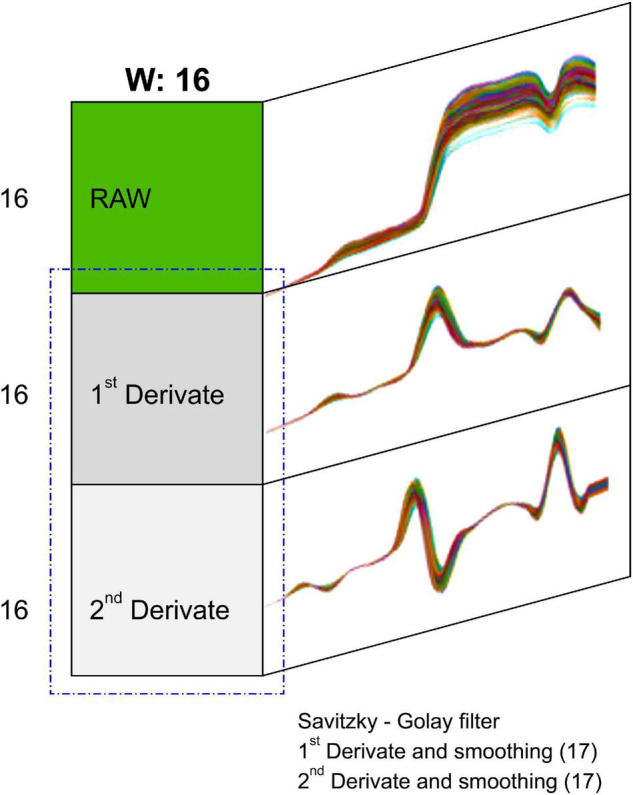
3D input layer structure expanded by a Savitzky–Golay filter.

If the noise and interference between channels are severe, preprocessing is performed on the spectral data, and essential tasks are performed to increase the significance of the data (Pei et al., 2019). The horizontal and vertical information of the 3D CNN hypercube data are 16 × 16, and preprocessing was performed to expand it by a factor of 3 to 16 × 48 into more in-depth nodes. A Savitzky–Golay filter (Savitzky and Golay, 1964) was used as the preprocessing algorithm, and a convolution coefficient of m equal to 17 was applied to 150 spectral data to perform smoothing (Figure 9). The first and second derivatives of these data were found, and the input was expanded in the vertical direction of the raw data. This was used in the same 3D CNN model, and the resulting accuracy was compared to the accuracy before expansion.

For the CNN model training, 70% of all data set were used for training, 10% were used for validation, and the remaining 20% were classified as a test set. Data classification was performed by a random split so that a specific strawberry leaf or image capturing condition was not learned. The models’ classification performance was compared based on accuracy; accuracy was also used as a statistical measure of how well the classification tests identified or excluded conditions. The accuracy corresponds to the proportion of correct predictions (both true positive and true negative) out of the total number of cases that were examined. The formula for quantifying accuracy is shown as follows.


(3) $$A\,c\,c\,u\,r\,a\,c\,y=\frac{T\,P+T\,N}{T\,P+T\,N+F\,P+F\,N}$$

where TP = True positive, FP = False positive, TN = True negative and FN = False negative.

## Results

### Correlations of the Three Local Area Classifications

**FIGURE 10:**
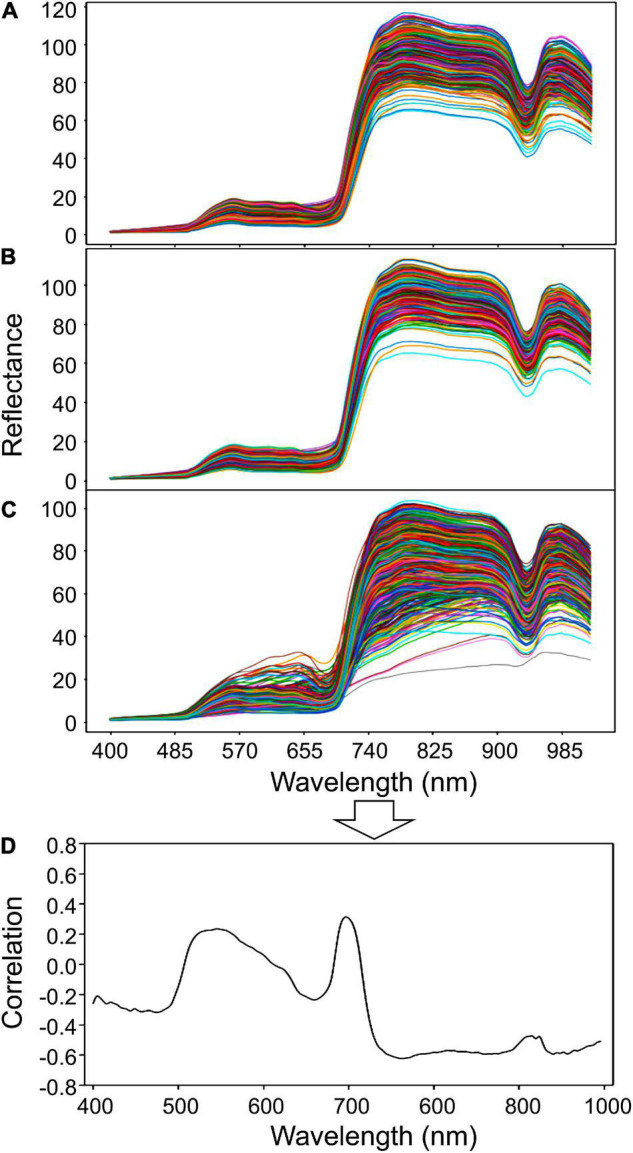
Wavelength graphs of **(A)** healthy areas, **(B)** asymptomatic areas, **(C)** infected areas and **(D)** graph of the correlations of each wavelength found through correlation analysis.

The wavelength bands of the three classes of healthy, asymptomatic, and infected areas were collected as shown in Figure 10, and the correlations of each of them were analyzed. The patterns of healthy and asymptomatic areas were similar; but in the 800–900 nm band, the maximum reflectance value of healthy areas was higher by about 5–10. In the infected areas, the curve of the wavelength was overall severe, and the reflectance was low due to light absorption. In the examination of the most effective wavelength band, the 760–800 nm wavelength band, which had a correlation coefficient of −0.6, was selected. The correlations were found to be not enough for creating early diagnosis sensors using filter images (through employing a multi-camera). Therefore, it is important to use the results of highly precise CNN prediction models that were developed using the entire hyperspectral sensor data wavelength band in order to increase the accuracy.

### Results of Comparing 2D Convolutional Neural Network Training and Classification Performance

**FIGURE 11:**
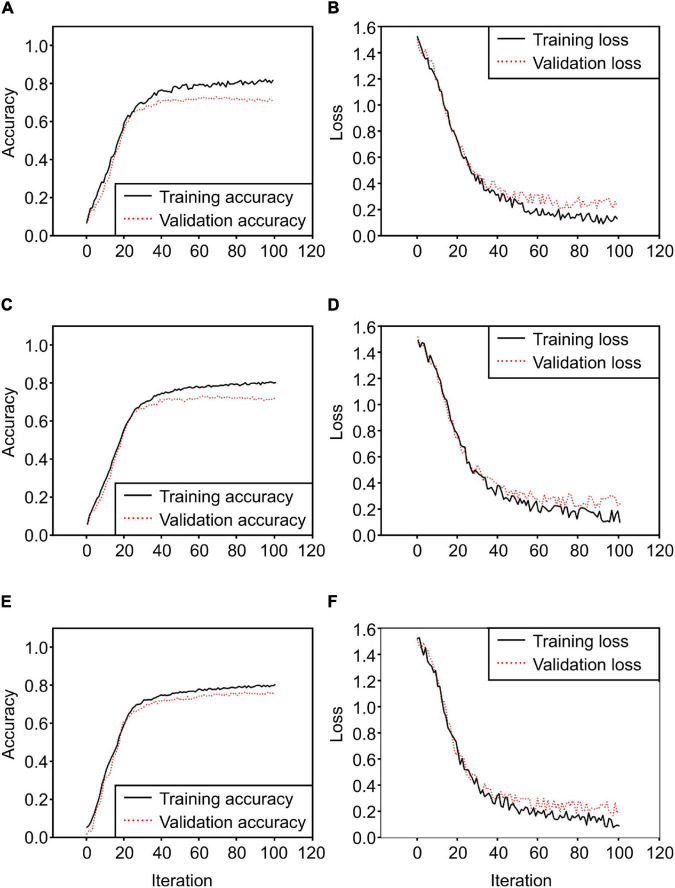
2D CNN model training results [**(A)** training accuracy of the 2D CNN model proposed in this study, **(B)** loss of the 2D CNN model, **(C)** accuracy of the 2D CNN model with the VGGnet-19 structure, **(D)** loss of the 2D CNN model with the VGGnet-19 structure, **(E)** accuracy of Resnet50, **(F)** loss of Resnet50].

**FIGURE 12:**
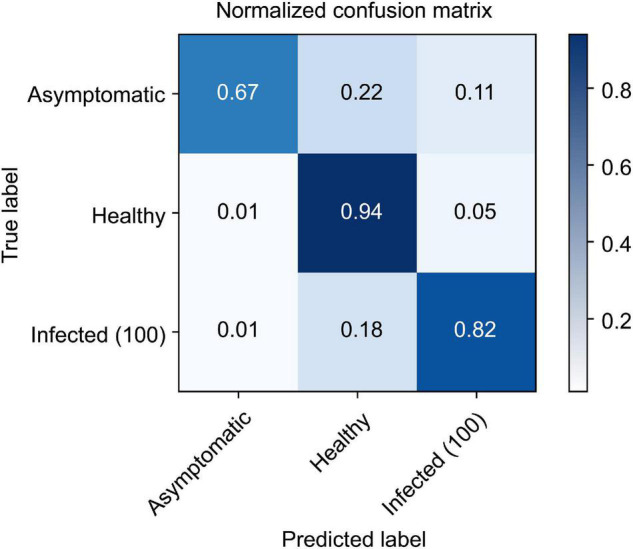
Confusion matrix results from the trained Resnet-50 model to infer the test set.

After converting the collected ROI 3D cubes into 2D cubes at a dimensionality of 256 × 150, classification models were developed using the three 2D CNN models. Each of them was trained for 100 iterations, and their loss and accuracy were recorded. The three models used the same Adam optimization algorithm, and categorical cross-entropy was selected as the cost function. First, the 2D model proposed in this study (Figure 5) had a training accuracy of 0.79 and a validation accuracy of 0.71 (Figure 11A); the change in loss at this time is shown in Figure 11B. As for the VGGnet-19 results, the changes in accuracy and loss can be seen in Figures 11C,D. The training accuracy that ultimately converged was 0.80, and the validation accuracy was 0.72. The model that used the Resnet-50 structure showed similar results. The changes in accuracy and loss can be seen in Figures 11E,F. The training accuracy was 0.81, and the validation accuracy was 0.74; these were the best results by a small margin. The accuracy of the three models converged at approximately 20–30 iterations, and the amount of time that each of them took to complete an iteration were 823, 1,544, and 966 ms, respectively. The Resnet-50 structure was the most efficient and showed the highest accuracy. Figure 12 shows a confusion matrix that was found by inferring a separately classified test set using Resnet-50, which had the highest accuracy. The total accuracy was 0.74, and the accuracies for asymptomatic, healthy, and infected areas were 0.67, 0.94, and 0.82, respectively. Thus, the accuracy for asymptomatic areas was somewhat low.

### 3D Convolutional Neural Network Training and Classification Performance Comparison Results

**FIGURE 13:**
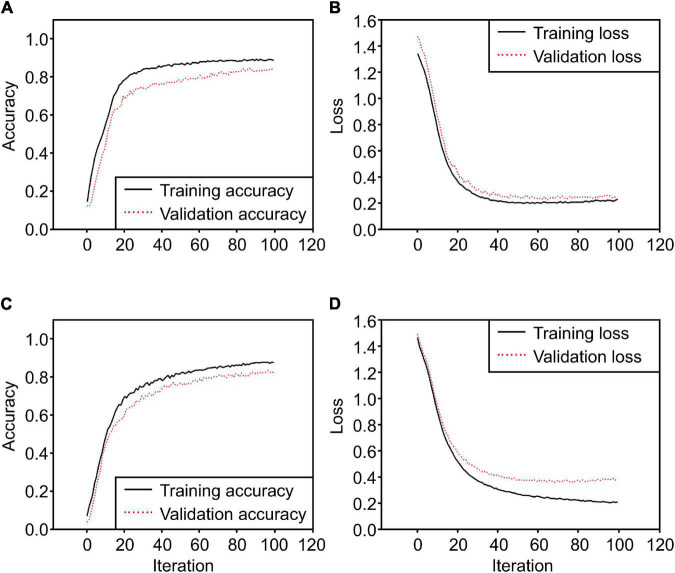
3D CNN model training results [**(A)** training accuracy of the 3D CNN model proposed in this study, **(B)** loss of 3D CNN model, **(C)** accuracy of 3D CNN model using extended input, **(D)** loss of 3D CNN model using extended input].

The 3D CNN that is proposed in this study uses a method that directly configures the layers of the generated hypercube data with a 3D filter. The 3D CNN model that uses 16 × 16 × 150 raw data as input was compared with the extended input 3D CNN model that preprocesses the raw data at 16 × 48 × 150 and adds two times the number of blocks. The results can be seen in Figure 13. The training accuracy of the 3D CNN using raw input was 0.87, and the validation accuracy was 0.83, while the training accuracy of the 3D CNN using extended input was ultimately 0.89, and the validation accuracy was 0.84. The results of the model that uses extended input were better, and when the training times were compared, the average times spent on an iteration by the two models were 15,240 and 110,055 ms, respectively. It was found that the training time spent by the extended 3D CNN was greater than that of the 2D CNN by more than a factor of 100 on average.

**FIGURE 14:**
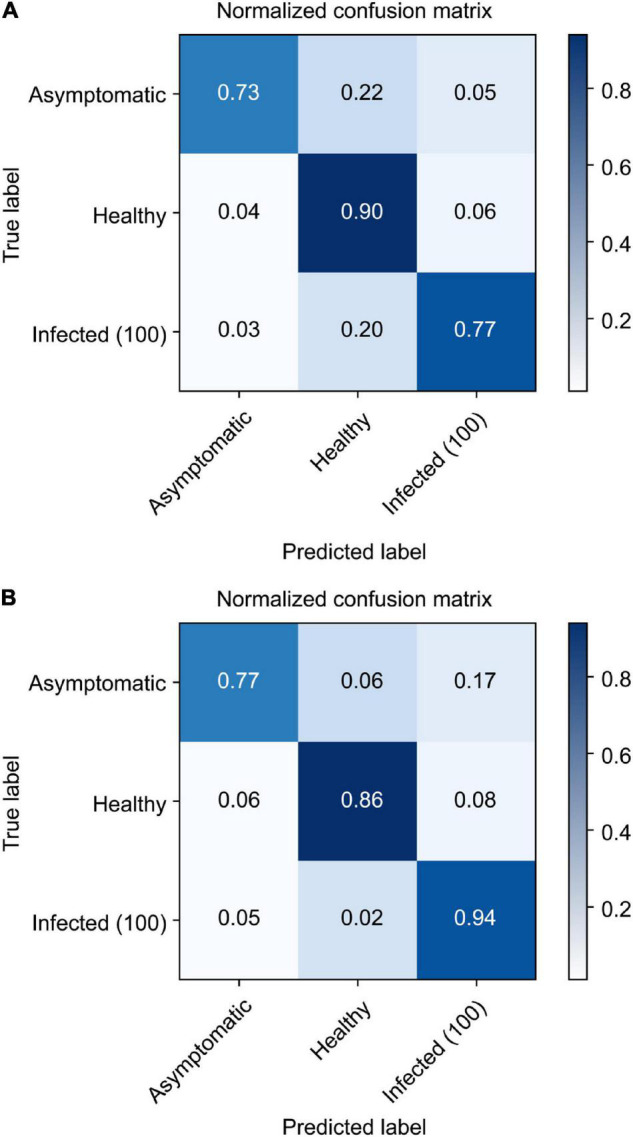
Confusion matrix results from the trained 3D CNN model to infer the test set [raw 3D CNN **(A)**; extended input 3D CNN **(B)**].

Figure 14 shows the accuracy for each class when the test samples were inferred *via* the two developed 3D CNN models. Asymptomatic infections are the classification goal of this study, and the two models obtained results of 0.73 and 0.77, respectively. This shows that they had higher classification accuracy than Resnet-50.

## Discussion

This study aimed to develop a classification model that uses hyperspectral imaging for early diagnosis of gray mold disease in strawberries and ultimately find an approach that can be used in practice. If effective wavelength bands are detected in the results shown in Figure 10, and this correlation has a high value, spectral imaging technology that can selectively measure small wavelength bands may be used; however, at a correlation of approximately 0.6, a deep learning based model is employed, and all of the hyperspectral data of each pixel must be used. However, the classification accuracy obtained by the 3D CNN model confirmed this possibility with a validation accuracy of 0.84, which is considered to be an adequate result.

A great deal of research has been performed on hyperspectral images using 1D and 2D CNNs, and it was necessary to search the effective image areas and wavelength bands in captured hyperspectral images in order to understand plant diseases (Xie et al., 2017; Hsieh and Kiang, 2020). This approach is efficient, but the feasibility of developing a model becomes very low if there are restrictions on the search for effective features. Therefore, it was necessary to develop 3D CNN models in order to use the 3D hyperspectral data without modification; this study has confirmed the potential feasibility of the proposed 3D CNN model in the field of hyperspectral images through a performance comparison with the 2D CNN Resnet and VGGnet models. Since the 3D CNN’s filter and layer configuration must be more complex, a great deal of computing power is needed for training; hence, it is difficult to perform training with 3D inputs that have large numbers of pixels when considering the memory that can be processed every time. Therefore, in previous studies, entire images of leaves (256 × 256 or more) could be made into single input layers when detecting diseases, but when using a 3D CNN, a local area must be cut out in order to be used. In the future, it will be necessary to conduct studies to drastically improve the computational speed of GPUs and computational 3D filters. If improvements are made in this area, increased performance can be expected from 3D CNNs not only in studies that use HSI, but also deep learning using continuous video frames and applications involving medical MRIs.

Hyperspectral imaging can be seen as a limitation in model development in a controlled environment, such as the high price of the device, controlled light condition, and location of samples. In this study, it is still necessary to study applicability such as configuration design of hyperspectral camera related to field application in the future in environments of strawberry greenhouses. The field applicability research considers the selective wavelength region of hyperspectral and requires the development of technology that can collect information in real-time. This clearly requires an initial capital investment to adopt the employed approach on a commercial scale (Taheri-Garavand et al., 2021a). Nevertheless, the wide-ranging large-scale commercial applications can provide high returns through considerable improvements in process enhancement and cost reduction.

**FIGURE 15:**
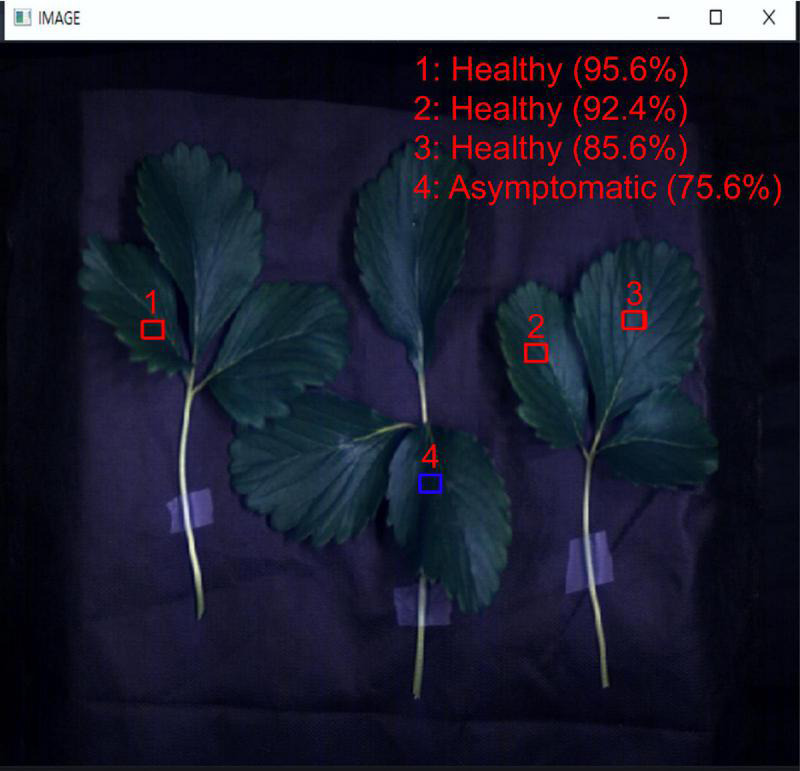
Program that loads hyperspectral images and diagnoses the infection state of the corresponding areas manually (through the user clicking on the mouse).

In this study, a local area value of 16 × 16 × 150 was proposed, and this size needs to be flexible depending on the equipment used in this study, the distance between samples, and the distribution of gray mold disease on strawberry leaves. Depending on the size of this cube, it can be used as a reference when applying to the field in the future or conducting diagnostic research on leaves and pests of other plant types. However, the use of a model that makes determinations based on local areas is possible through the following program. Figure 15 shows a program that uses the developed 3D CNN model and the results for areas that have been automatically selected by the model as the user examines RGB hyperspectral images and moves the mouse to click and select the desired areas. This is more specific than scanning the entire leaf and determining its state, and it has been found to be helpful in finding asymptomatic areas in strawberry plants that are infected with gray mold disease. The collected strawberry gray mold disease classification information that was developed *via* supplementary data and the developed model will be shared so that it can be used by other researchers in the field.

## Conclusion

This study has proposed the use of hyperspectral imaging to classify gray mold disease on strawberry leaves and developed a model for classifying healthy, asymptomatic, and unhealthy areas. Three 2D CNN models with different structures were compared, and the results showed that the Resnet-50 model had a training accuracy of 0.81 and a validation accuracy of 0.74. Our research proposed a 3D CNN model that can use the input structure of hyperspectral data without modification, and the results showed that the model that used cube data with a dimensionality of 16 × 16 × 150 had a training accuracy of 0.87 and a validation accuracy of 0.83. In addition, a 3D CNN that used extended input ultimately had a training accuracy of 0.89 and a validation accuracy of 0.84.

In conclusion, in this study, the development of a 3D CNN model for diagnosing gray mold disease of strawberries in a non-contact and non-destructive way using hyperspectral images, the learning data acquisition process, and data processing technology required for this, were introduced, and asymptomatic infection sites were reliably identified. Detection is expected to be utilized as a diagnostic technology that can determine the onset of gray mold disease at a relatively early stage in actual farms in the future. Accordingly, as a final result, a diagnostic program that can accurately predict gray mold disease on strawberry leaves at the local site level is proposed.

## Data Availability Statement

The raw data supporting the conclusions of this article will be made available by the authors, without undue reservation.

## Author Contributions

SHP and TSL: conceptualization. H-YK and SHP: funding acquisition. D-HJ and JDK: investigation. D-HJ, JDK, and TSL: methodology. H-YK: project administration. D-HJ: software. HSK: validation. D-HJ and SHP: writing original draft. HSK and SHP: writing review and editing. All authors have read and agreed to the published version of the manuscript.

## Conflict of Interest

The authors declare that they have no known competing financial interests or personal relationships that could have appeared to influence the work reported in this manuscript.

## Publisher’s Note

All claims expressed in this article are solely those of the authors and do not necessarily represent those of their affiliated organizations, or those of the publisher, the editors and the reviewers. Any product that may be evaluated in this article, or claim that may be made by its manufacturer, is not guaranteed or endorsed by the publisher.
